# Targeting the leukemic bone marrow microenvironment

**DOI:** 10.18632/oncotarget.22103

**Published:** 2017-10-27

**Authors:** Eva Kristin von der Heide, Martin Neumann, Claudia D. Baldus

**Affiliations:** Claudia D. Baldus: Charité Campus Benjamin Franklin, Department of Hematology and Oncology, Hindenburgdamm, Berlin, Germany

**Keywords:** mesenchymal stromal cells, acute myeloid leukemia, microenvironment, molecular characterization, next generation sequencing

Alterations of the bone marrow (BM) microenvironment, such as aberrant growth signals, are contributing factors in hematopoietic malignancies. In leukemia, transformed blasts induce morphological changes in the niche anatomy and stromal-leukemia interactions promote disease progression (reviewed in [1]). However, molecular alterations in primary human niche cells leading to functional impairment of hematopoietic stem and progenitor cell (HSC/HSPC) support have not yet been fully defined. A detailed characterization of primary human BM mesenchymal stromal cells (BM-MSC) derived from leukemia patients will unravel specific alterations and disclose their role in induction and maintenance of the disease.

In these lines, we recently reported a comprehensive molecular portrait of primary BM-MSC from acute myeloid leukemia (AML) patients using genome wide high throughput platforms and uncovered alterations on genetic, epigenetic and transcriptional levels in a multi omics approach [2]. Our analyses gave broad insights into a plethora of molecular alterations for a large cohort of AML BM-MSC and demonstrated a higher mutational burden in AML BM-MSC compared to healthy controls. However, no recurrent mutations were identified in our cohort.

The potential of a genetically altered microenvironment to induce hematopoietic disorders has yet exclusively been studied and demonstrated in mouse models. In humans, leukemic transformation as an immediate consequence of primary genetic alterations in niche cells remains elusive. A recent report discussed proinflammatory signals from the microenvironment in a mouse model for myelodysplastic syndrome (MDS) and Schwachman-Bodium-Diamond syndrome (SDS), both preleukemic conditions, as a driver for genotoxic stress in HSCs and HSPCs. *SDBS* deletion in niche cells induced mitochondrial dysfunction, oxidative stress and DNA damage response in HSPCs [3]. Indeed, the increased mutational burden in AML BM-MSC samples indicates a higher susceptibility for AML BM-MSC to acquire genetic alterations [2], but putative disease-initiating mutations in human niche cells remain to be uncovered. This also requires additional genetic screening of human stromal cell subpopulations.

On DNA methylation and transcriptional levels, our comprehensive molecular portrait revealed deregulation of adhesion molecules, extracellular matrix components as well as altered metabolic pathways in AML BM-MSC compared to healthy controls [2]. Metabolic adaptation is a key mechanism in the complex interplay between malignant and stromal cells. Altered nutrient supply has already been described for the solid tumor microenvironment (reviewed in [4]). As identified in our genome wide characterization, imbalances in stromal metabolism might alter nutrient uptake of leukemic cells and fuel malignant cell growth. Recently, Ye et al. demonstrated the adaption of a leukemic stem cell (LSC) population and utilization of gonadal adipose tissue as a niche to support their metabolism and evade chemotherapy [5]. Another study showed increased basal glycolysis in leukemia mediated by direct contact to stromal cells in a co-culture. This metabolic adaption was driven by the CXCL12/CXCR4/mTOR axis indicating a role of stromal CXCL12 in a glycolytic shift and Warburg effect promotion with the consequence of chemotherapy resilience in AML [6]. This exploits the efficacy of CXCL12/CXCR4 signaling abrogation in AML treatment and sheds light on the therapeutic capacity of targeting the metabolic adaptions of both, the microenvironment and the sheltered malignant cell.

Exploration of stroma-mediated chemoresistance mechanisms will promote the identification of potential targets for niche-directed therapies to overcome drug resistance and eradicate LSCs. The data suggested significant deregulation of adhesion molecules that could serve as interaction sites for direct cell-to-cell contact [2]. Residing in close contact to niche cells, LSCs evade from apoptosis induction upon chemotherapy exposure. A recent study presented a reverse phase protein array based profile of 53 key molecules and unraveled stroma-mediated deregulation of signaling pathway networks in response to inhibitors such as temsirolimus (mTOR), ABT737 (BCL2/BCL-XL), and Nutlin-3a (MDM2). This study demonstrated an activation of Akt in singularly ABT737 or Nutlin-3a treated leukemia samples mediated by stromal contact and an evidence for the efficacy of combinational treatment to overcome stroma-mediated chemoresistance [7].

Future molecular profiling of leukemic niche cells certainly demands detailed analyses of sorted primary stromal subpopulations to eliminate potential *in vitro* expansion bias and to explore the disease driving potential of each individual stromal subpopulation in hematopoietic malignancies. For instance, profiling of leukemic stromal subpopulations of mesenchymal and non-mesenchymal origin, such as osteoblasts and macrophages, respectively, would help to composite the picture of a leukemic microenvironment. This, in turn, requires a rigorous definition of an immature mesenchymal progenitor as well as detailed exploration of mesenchymal progeny in context of the human HSC niche. Hu et al. discussed a Sca1+ murine mesenchymal stromal cell population as being a common mesenchymal progenitor [8]. The diversity of already described stromal markers demonstrates the urgent need of defining specific marker panels and sorting strategies for human mesenchymal progenitors.

Taken together, our data and other studies revealed alterations in the leukemic BM microenvironment thereby offering a platform for the identification of putative targets for niche directed therapies. The dynamic and diversity of cell-to-cell communication go beyond blast infiltration and macroscopic changes in the BM microenvironment and involve leukemia-mediated adaptions of niche components.
